# Evaluation of Chloropicrin as a Soil Fumigant against *Ralstonia solanacarum* in Ginger (*Zingiber officinale* Rosc.) Production in China

**DOI:** 10.1371/journal.pone.0091767

**Published:** 2014-03-11

**Authors:** Liangang Mao, Qiuxia Wang, Dongdong Yan, Taotao Ma, Pengfei Liu, Jin Shen, Yuan Li, Canbin Ouyang, Meixia Guo, Aocheng Cao

**Affiliations:** 1 Department of Pesticides, Institute of Plant Protection, Chinese Academy of Agricultural Sciences, Ministry of Agriculture, Beijing, People's Republic of China; 2 State Key Laboratory for Biology of Plant Disease and Insect Pests, Beijing, People's Republic of China; U. S. Salinity Lab, United States of America

## Abstract

**Background:**

Chloropicrin (Pic) offers a potential alternative to methyl bromide (MB) against *Ralstonia solanacarum* in ginger (*Zingiber officinale* Rosc.) production. MB is scheduled to be withdrawn from routine use by 2015 in developing countries.

**Methods:**

Pic treatments were evaluated in a laboratory study and in three commercial ginger fields.

**Results:**

Laboratory studies showed that the EC_50_ value and EC_80_ value of Pic were 2.7 and 3.7 mg a.i. kg^−1^ soil, respectively. Field trials in highly infested soil revealed that treatments of Pic at the dose of 50 g m^−2^ covered with totally impermeable film (TIF) or polyethylene film (PE) sharply reduced *Ralstonia solanacarum* and maintained high ginger yields. Both of the Pic treatments provided results similar to, or in some cases slightly lower than, MB with respect to *Ralstonia solanacarum* control, plant survival, plant growth and yield. All of the fumigant treatments were significantly better than the non-treated control.

**Conclusions:**

The present study confirms that the Pic is a promising alternative with good efficacy against *Ralstonia solanacarum* for ginger production and could be used in integrated pest management programs in China.

## Introduction

In 2011 the total ginger production in China, the second largest ginger (*Zingiber officinale* Rosc.) producing country in the world, was 426,032.00 tonnes (t) and the area harvested was 36,007.00 hectare (ha) [1]. Due to successive monocropping of ginger, *Ralstonia solanacarum*, which was originally described by Smith (1896) as the causative agent of bacterial wilt of solanaceous plants [2], has the ability to reduce yields greatly in the cultivation of ginger in China. At present, methyl bromide (MB) is widely used in ginger cultivation in China as a preplant soil fumigant against *R. solanacarum*. MB, however, has to be phased out by 1 January 2015 in developing countries owing to its detrimental effects on the stratospheric ozone layer [3]. The withdrawal of MB from use as an agricultural fumigant has prompted a large amount of research aimed at finding effective and economically acceptable alternatives [4].

The pesticides currently applied against *R. solanacarum* in ginger in China include fumigants and other chemical or biological pesticides, such as chloropicrin (Pic), cupric hydroxide, and *Bacillus cereus*. Pic is becoming one of the most important fumigants for ginger production in China. There is much information in the literature on the ability of Pic to control various soilborne pests effectively in many crops, for example tomatoes [5]–[9], strawberries [10], peppers [11]–[13] and others. However, little information has been reported on Pic against *R. solanacarum* in ginger production.

The present work was initiated to test the effects of Pic on *R. solanacarum* in laboratory studies. In addition, three field trials were carried out to evaluate two different Pic treatments as a potential alternative to MB against *R. solanacarum* for ginger production in China.

## Materials and Methods

### Laboratory studies

The dose-responses of *R. solanacarum* to different Pic concentrations were studied in the laboratory. With the authorization of the institute of Fangshan agricultural science, Beijing, soil samples in the laboratory studies were collected from the top 20 cm of greenhouse soil in Nanhe village, Dashiwo town, Fangshan district, southwest of Beijing. The field studies site does not involve endangered or protected species and the GPS coordinates are 39°33′9.3″N, 115°49′34.1″E. The soil was composed of 61.48% sand, 37.20% silt, and 1.32% clay, with organic matter content 25.38 g kg^−1^ soil and pH 6.49. The soil was sieved through a 2-mm mesh, and then mixed together. The soil moisture was 12.37% (w/w). Particle size analyses were performed using the pipette method [14]. The organic carbon content was determined by wet oxidation using the method of Walkley and Black [15]. The pH was measured in a 1:2.5 soil to H_2_O extract. Soil moisture content was measured in the drying oven at 105±5°C until mass constancy was achieved [16]. The soil was sterilized at 121°C for 30 mins using the high-pressure steam sterilization pot. *R. solanacarum*, was grown on triphenyltetrazolium chloride (TTC) selective media and then quantitatively incorporated into the sterilized soil.

300 g *R. solanacarum*-infested soil was placed into each of 0.5 L glass jars [17]–[19]. The following treatments were applied with three replicates: Pic alone (1.0, 2.0, 4.0, 8.0, 16.0, 32.0, 64.0, 128.0, 256.0 mg a.i. kg^−1^ soil) and non-treated control. Pic was injected into the soil by pipette (Eppendorf, Germany) and then the jars were immediately sealed with covers. The jars were placed in incubators at 28°C for 7 days. The jars were opened to release the residual fumigant for a day and *R. solanacarum* was isolated from the soil quantitatively based on the method described by Kelman [20].

### Field trials

In 2012 and 2013, with the authorization of Laiwu agriculture bureauthe, Shandong Province, three field experiments were conducted in three ginger fields located in Xiaoxia village, Zhaili town, Laiwu city, Shandong province, China (trial I: 36°17′31.9″N, 117°28′19.6″E), Hanwangxu village, Zhaili town, Laiwu city, Shandong province, China (trial II: 36°17′52.1″N, 117°26′07.4″E), and Caodaxia village, Zhaili town, Laiwu city, Shandong province, China (trial III: 36°16′30.8″N, 117°30′05.1″E), respectively. The field studies site does not involve endangered or protected species. And there are no specific permissions required for the field sites. All field sites are located in intensive ginger production areas of Laiwu, a region which has a long history of ginger production. However, *R. solanacarum* has become a big problem due to the continuous cropping. The major soil-borne disease is plant death caused by *R. solanacarum*. Details relevant to these trials are given in **Table 1** and **2**.

**Table 1:** Soil characteristics in the experimental sites.

Site	Soil moisture (%)	pH (1:2.5)	Organic matter (g kg^−1^)	N/NH_4_^+^ (mg kg^−1^)	N/NO_3_^−^ (mg kg^−1^)	Available K (mg kg^−1^)	Available P (mg kg^−1^)
trial I	14.95	6.78	22.36	38.37	71.09	341.54	392.11
trial II	14.09	6.90	14.17	18.31	43.35	239.56	231.26
trial III	18.20	6.16	17.40	47.42	77.50	479.21	402.20

**Table 2:** Relevant trial dates and other details.

Site	Fumigant application	Tarp removal	Ginger transplant	Germination investigation	Plant growth evaluation^a^	End of the trial	Preceding crop
trial I	02/11/12; 11/03/13	09/04/13	19/04/13	26/06/13	07/08/13	13/10/13	Ginger
trial II	03/11/12; 12/03/13	10/04/13	22/04/13	27/06/13	08/08/13	13/10/13	Ginger
trial III	03/11/12; 12/03/13	10/04/13	23/04/13	27/06/13	08/08/13	14/10/13	Ginger

a Plant growth evaluation included ginger plant height and number of leaves per tiller.

The following fumigant products were used in the study: (a) Pic 99.5 LD (Dalian Dyestuffs & Chemicals Co., China), a commercial liquid product containing 99.5% Pic; (b) MB 98 TC (Lianyungang Dead Sea Bromine Co. Ltd, Jiangsu Province, China) containing 98% MB and 2% chloropicrin. The soil mulches were 0.04 mm polyethylene film (PE) (Baoding Baoshuo Plastic Co., Ltd., Hebei Province, China) and 0.04 mm totally impermeable film (TIF) (Jiahe Industrial Co., Ltd., Jiangsu Province, China).

**Figure 1:**
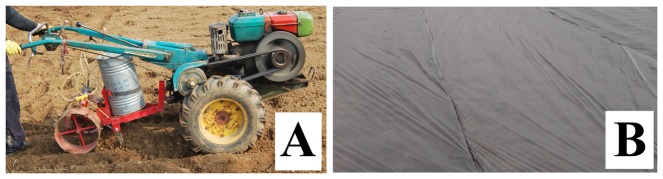
Application of Pic in field trials: (A) a manual injection machine was used to apply Pic in the soil; (B) the treatments were covered with PE or TIF film.

Each Pic treated plot area was designed to 50 m^2^. Each MB treated plot area and untreated control plot area are both designed to 25 m^2^. The treatments were designed as randomized blocks with three replicates (**Table 3**). The tested treatments were MB, used as a reference treatment, Pic, and untreated control. Pic liquid was injected into the soil at 15 cm depth via a manual injection machine at a rate of 50 g m^−2^ (**Figure 1**). The Pic treatments were applied in two different seasons: one was applied in November 2012 (soil temperature at 5 cm depth was about 15 to 21°C during the day) and covered with TIF film; another was applied in March 2013 (soil temperature at 5 cm depth was about 13 to 19°C during the day) and covered with PE film (**Figure 1**). MB was applied by using evaporating iron jars under a PE sheet and allowing it to vaporize in situ (cold method) at a rate of 40 g m^−2^ in March 2013, and a small arc tunnel (40 cm height) was constructed under the mulch with the aim of improving the fumigant distribution [21]. The ginger plants were 40 cm apart, planting distance 25 cm, about 6500 plants per mu. Traditional cultivation techniques were used in all plots.

**Table 3:** Experimental program of the field trials.

Trial	Fumigant and formulation^a^	Rate (g a.i. m^−2^)	Tarp^b^	Application method	Fumigation date	Abbreviation used for the treatment
trial I	Pic 99 LD	50	TIF	Injection	02/11/2012	Pic 50 TIF
	Pic 99 LD	50	PE	Injection	11/03/2013	Pic 50 PE
	MB 98 TC	40	PE	Cold method	11/03/2013	MB 40 PE
	Untreated control	−	−	−	02/11/2012	
trial II	Pic 99 LD	50	TIF	Injection	03/11/2012	Pic 50 TIF
	Pic 99 LD	50	PE	Injection	12/03/2013	Pic 50 PE
	MB 98 TC	40	PE	Cold method	12/03/2013	MB 40 PE
	Untreated control	−	−	−	03/11/2012	
trial III	Pic 99 LD	50	TIF	Injection	03/11/2012	Pic 50 TIF
	Pic 99 LD	50	PE	Injection	12/03/2013	Pic 50 PE
	MB 98 TC	40	PE	Cold method	12/03/2013	MB 40 PE
	Untreated control	−	−	−	03/11/2012	

a Abbreviations: Pic  =  chloropicrin; MB  =  methyl bromide; TC  =  Technical.

b Abbreviations: PE  =  polyethylene film; TIF  =  totally impermeable film.

Soil *R. solanacarum* populations [colony-forming units (cfu) g^−1^ soil] were determined after fumigation from soil at the depth of 0–20 cm. Soil from each plot was sampled from 3 spots along the diagonal lines in a plot. *R. solanacarum* was determined using the same methods as the **Laboratory studies**.

Ginger germination was evaluated at 9 WAT (weeks after transplant). Plant growth (ginger plant height, tillers/plant and mortality) were evaluated at 15 WAT (weeks after transplant) (20 plants per plot). The yield, plant height and root disease severity in ginger were evaluated at the end of the trials. Twenty ginger plants were picked from each plot, and the severity of ginger root disease was assessed separately, based on a disease severity scale of 0–4, where 0  =  healthy plant and root, without disease; 1  =  black brown rotten roots comprising <25% of the entire root system; 2 = 26–50%; 3 = 51–75%; and 4 = 76–100% black brown rotten roots [22].

### Statistical analyses

#### Laboratory studies

The bacterial control efficacy of treatments can be calculated according to the following equation.
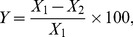
(1)where *Y* is the control efficacy on bacteria, *X_1_* is the bacteria population of the untreated control, *X_2_* is the bacteria population of fumigant treatments.

The experimental design consisted of a randomized complete block with three replications. A nonlinear dose-response curve, which was analyzed with Origin (Origin Pro 8.0 for Windows), was used to describe the relationship between the *R. solanacarum* mortality (*y*) and the logarithm of the concentration of fumigant (*x*):
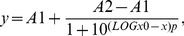
(2)


#### Field trials

The control efficacy on bacteria was calculated using the same equation as the *Laboratory studies*.

The disease scores recorded for each plot were converted into disease indices (% *DI*) using the formula described by McKinney [22]

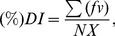
(3)where ƒ  =  number of plants in each class, *v*  =  class value, *N*  =  number of observed plants, and *X*  =  highest value in the evaluation scale.

Data were analyzed for ANOVA with SAS (SAS, version 8.0 for Windows). Significant differences among means were determined by Fisher's LSD test at *P* = 0.05 [23], [24]. Data for soil bacteria populations were transformed as necessary (square root transformations for small numbers [<100] and log10 for large numbers [> 100] for statistical analyses), but all data are reported as non-transformed values. The data in percentages (mortality and root disease index) were normalized with arcsine square root transformation prior to ANOVA.

## Results

### Laboratory studies

**Figure 2:**
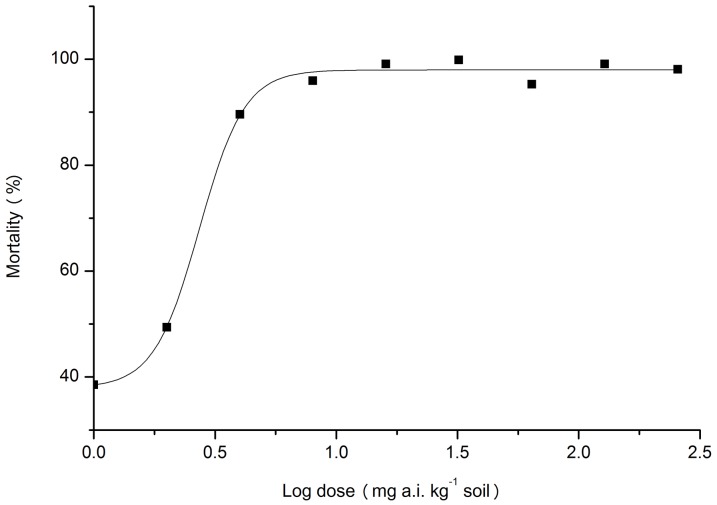
Response of *R. solanacarum* to different Pic concentrations after fumigation of a sandy loam soil for 7 days at 28°C.

The nonlinear dose-response curve of *R. solanacarum* to different concentrations of Pic indicated a strong relationship between increases in fumigant concentration and mortality, with *R* ^2^ values≥0.994 (**Figure 2**). A nonlinear curve fit model was expressed as follows:
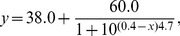
(4)


The nonlinear curve fit (DoseResp) revealed that the concentrations required to control 50% (EC_50_) and 80% (EC_80_) of *R. solanacarum* were 2.7 and 3.7 mg a.i. kg^−1^ soil, respectively.

### Field trials

#### Trial I

**Figure 3:**
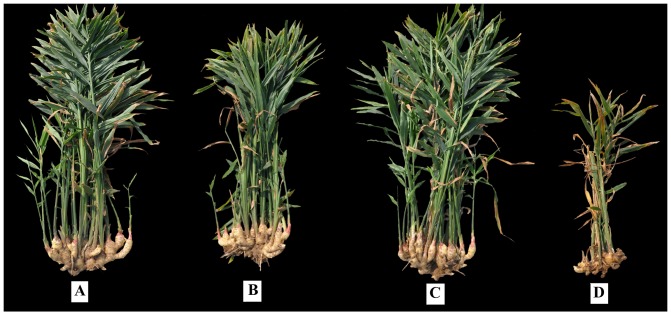
Ginger plants grown in plots treated with (A) Pic 50 TIF, (B) Pic 50 PE, (C) MB 50 PE and (D) Untreated control at the end of trials, respectively (trial I).

The untreated controls in trial I were heavily infested by *R. solanacarum* (**Table 4** **and** **Figure 3**). *R. solanacarum* levels were significantly lower in the chemical treatments compared with the untreated control, except for Pic 50 PE treatments applied in March. In trial I the greatest reduction of *R. solanacarum* was provided by MB 40 PE treatments (94.92%), followed by Pic 50 TIF treatments (85.27%) and Pic 50 PE treatments (75.93%) (**Table 4**).

**Table 4:** Effects of soil fumigation on colony-forming units (cfu) of *R. solanacearum* on selective media of 1 g from soil after fumigation.

Site	Treatment^a^	*R. solanacearum*	
		cfu/g^a^	% reduction
trial I	Pic 50 TIF	947b	85.27
	Pic 50 PE	1547ab	75.93
	MB 40 PE	327b	94.92
	Untreated control	6427a	
trial II	Pic 50 TIF	1093b	84.25
	Pic 50 PE	1667b	75.98
	MB 40 PE	293c	95.77
	Untreated control	6940a	
trial III	Pic 50 TIF	340bc	93.82
	Pic 50 PE	583b	89.39
	MB 40 PE	267c	95.15
	Untreated control	5553a	

a In each column, data are means of three replication. Means followed by the same letter are not different (*P* = 0.05) according to the LSD test.

Ginger germination levels in all treatments were at least 96.97% at 9 WAT, and there was no significant difference between the chemical treatments and the untreated control at this stage (**Table 5**). The fumigation treatments significantly affected plant height, number of leaves per tiller and plant mortality at 15 WAT (**Table 5**). In trial I the ginger grown in MB PE treatment plots had the greatest plant height (91.0 cm), the largest number of leaves per tiller (15) and the lowest plant mortality (0.00%) at 15 WAT. However there was no significant difference between the three fumigant treatments with respect to plant mortality (which ranged from 0.0% to 0.5%), and all fumigant treatments substantially reduced plant mortality compared to the untreated control (60% mortality) (**Table 5**).

**Table 5:** Effect of fumigation treatments on germination and plant growth during the ginger crop growth period.

Site	Treatment	Germination (%)	Plant height (cm)	Number of leaves/tiller^b^	Mortality (%)
trial I	Pic 50 TIF	99.56a^a^	80.0ab	11b	0.44b
	Pic 50 PE	99.49a	78.3b	15ab	0.51b
	MB 40 PE	98.10a	91.0a	15a	0.00b
	Untreated control	96.97a	61.7c	5c	60.00a
trial II	Pic 50 TIF	98.00a	87.3a	15a	0.00b
	Pic 50 PE	98.46a	85.7a	15a	0.51b
	MB 40 PE	100.00a	84.7a	16a	0.00b
	Untreated control	97.76a	62.7b	5b	86.55a
trial III	Pic 50 TIF	98.56ab	88.7a	13b	0.44b
	Pic 50 PE	97.44ab	83.7a	17a	1.03b
	MB 40 PE	100.00a	82.7a	14ab	0.00b
	Untreated control	88.10b	57.3b	3c	70.85a

a In each column, data are means of three replication. Means followed by the same letter are not different (*P* = 0.05) according to the LSD test.

b Number of leaves/tiller were counted from the one who had the most leaves in the all tillers of one ginger plant.

Compared to the untreated control, fumigation treatments significantly affected plant height, number of tillers per plant, root disease index and yield at the end of the trials (**Table 6**). Pic 50 TIF treatments produced the greatest plant height (99.8 cm) and the lowest root disease index (0.00%), followed by MB 40 PE and Pic 50 PE. Pic 50 TIF and MB 40 PE treatments showed no significant difference in plant height and root disease index (**Table 6**). There was no significant difference in the number of tillers per plant in all chemical treatments. Ginger grown in the MB 40 PE plots had the lowest plant mortality (0.00%), followed by Pic 50 TIF and Pic 40 PE treatments, but there was no significant difference in plant mortality between MB 40 PE and Pic 50 TIF treatments (**Table 6**). Ginger grown in the untreated plots had the lowest yield (1.50 Kg m^−2^). The plots treated with MB 40 PE provided the highest yield (6.69 Kg m^−2^), but this was not statistically different to the two Pic treatments (**Table 6**).

**Table 6:** Effect of fumigation treatments on ginger plant height, tillers/plant, mortality, root disease, and yield at the end of the trials.

Site	Treatment	Plant height (cm)	Tillers/plant	Mortality (%)	Root disease index (%)	Yield (Kg m^−2^)
trial I	Pic 50 TIF	99.8a^a^	15a	1.25c	0.00c	6.27a
	Pic 50 PE	81.9b	14a	10.00b	26.88b	6.20a
	MB 40 PE	97.9a	16a	0.00c	1.88c	6.69a
	Untreated control	24.9c	2b	88.94a	93.13a	1.50b
trial II	Pic 50 TIF	95.0a	15a	8.50b	12.50b	7.29a
	Pic 50 PE	87.7a	13a	10.55b	12.50b	5.32b
	MB 40 PE	98.4a	15a	0.00b	0.00b	6.24ab
	Untreated control	0.0b	0b	100.00a	100.00a	0.00c
trial III	Pic 50 TIF	90.6a	11b	10.00b	15.63b	4.99b
	Pic 50 PE	93.1a	15ab	5.00b	6.25b	5.49ab
	MB 40 PE	98.0a	17a	0.00b	0.00b	7.31a
	Untreated control	42.2b	6c	90.85a	78.13a	1.87c

a In each column, data are means of three replication. Means followed by the same letter are not different (*P* = 0.05) according to the LSD test.

#### Trial II

The untreated controls in trial II were also heavily infested by *R. solanacarum* (**Table 4**). All chemical treatments reduced *R. solanacarum* at least 75.98%. The lowest level of *R. solanacarum* of was provided by MB 40 PE treatments, followed by Pic 50 TIF and Pic 50 PE treatments. However there was no significant difference in *R. solanacarum* levels between the two different Pic treatments (**Table 4**).

Ginger germination levels in all treatments were at least 97.76% at 9 WAT, and there was no significant difference at this stage (**Table 5**). Fumigation treatments significantly affected plant height, number of leaves per tiller and plant mortality at 15 WAT (**Table 5**). Ginger grown in the untreated controls had the lowest plant height (62.7 cm), the least number of leaves per tiller (5) and the highest plant mortality (86.55%) at 15 WAT. Among the three fumigation treatments there was no statistical difference in plant height, number of leaves per tiller and plant mortality at 15 WAT, and all were significantly different to the control (**Table 5** **)**.

At the end of trial II, the fumigation treatments significantly affected plant height, number of tillers per plant, root disease index and yield (**Table 6**). Plant height was significantly greater in all chemical treatments compared with the untreated control. The greatest plant height was observed in plots treated with MB 40 PE (98.4 cm), followed by Pic 50 TIF and Pic 50 PE. However, there was no significant difference in plant height between the Pic and MB treatments. Similarly, the plots treated with MB 40 PE provided the greatest number of tillers per plant (15), the lowest ginger mortality (0.00%), and the lowest ginger root disease index (0.00%), but these results were not statistically different from the Pic treatments.

Ginger grown in the untreated plots had the lowest yield (0.00 Kg m^−2^) (**Table 6**). The plots treated with Pic 50 TIF provided the highest yield (7.29 Kg m^−2^), and this was not statistically different to the yield obtained with MB 40 PE but was significantly higher than the yield obtained with the Pic 50 PE treatment (**Table 6**).

#### Trial III

Trial III also found that the untreated controls were heavily infested by *R. solanacarum* (**Table 4**). All chemical treatments reduced *R. solanacarum* at least 89.39%. The lowest level of *R. solanacarum* was also provided by MB 40 PE treatments, followed by Pic 50 TIF and Pic 50 PE treatments. However, there was no significant difference in *R. solanacarum* levels between Pic 50 TIF and MB 40 PE treatments (**Table 4**).

At 9 WAT there was no significant difference in ginger germination levels between the treatments except that the levels in MB 40 PE treatments (100%) were significantly higher than the untreated controls (88.10%) (**Table 5**). Fumigation treatments significantly affected plant height, number of leaves per tiller and plant mortality at 15 WAT (**Table 5**). Ginger grown in the untreated controls had the lowest plant height (57.3 cm), the least number of leaves per tiller (3) and the highest plant mortality (70.85%) at 15 WAT. The three fumigation treatments showed no significant difference in plant height, number of leaves per tiller and plant mortality (**Table 5**).

At the end of the trials, fumigation treatments significantly affected plant height, number of tillers per plant, root disease index and yield (**Table 6**). Plant height was significantly greater in all chemical treatments compared with the untreated control. The greatest plant height was observed in plots treated with MB 40 PE (98.0 cm), followed by Pic 50 PE and Pic 50 TIF, however there was no significant difference between the chemical treatments. Similarly, the plots treated with MB 40 PE provided the lowest ginger mortality (0.00%) and the lowest ginger root disease index (0.00%), but this was not statistically different from the Pic treatments. Ginger grown in the untreated plots had the lowest number of tillers per plant (6) and lowest yield (0.00 Kg m^−2^) (**Table 6**). The plots treated with MB 40 PE provided the greatest number of tillers per plant (17) and the highest yield (7.31 Kg m^−2^); this was not statistically different from the yield obtained with Pic 50 PE but was significantly higher than the yield obtained with Pic 50 TIF (**Table 6**).

## Discussion

After three to five years' successive monocropping of ginger, ginger bacterial wilt caused by soil-borne *R. solanacarum* becomes greater and greater in China and the ginger producers have to plant other vegetables such as Chinese onion (*Agrimonia fistulosum* L.), garlic (*Agrimonia sativum* L.), Chinese cabbage (*Brassica campestris* L. spp.), radish (*Raphanus sativus* L.), maize (*Zea mays* L.) and others. Ginger has a relatively high economic value compared to other vegetables, so the application of a fumigant is regarded as an essential practice to protect ginger plants from soil-borne *R. solanacarum* in China. In our research, laboratory studies and three field trials were conducted to determine effects of several Pic treatments on *R. solanacarum* in ginger production.

Our laboratory studies showed that Pic can provide excellent control of *R. solanacarum* after fumigation of a sandy loam soil for 7 days at 28°C. The specific mechanism by which Pic controls *R. solanacarum* was not examined, however, the current laboratory results indicated that Pic is feasible and effective in controlling *R. solanacarum.*

In the present field trials, both Pic treatments (Pic 50 TIF applied in November, and Pic 50 PE applied in March) sharply reduced the colony-forming units (cfu) of *R. solanacarum* on media, and maintained high ginger yields in commercial production. Based on the present field results, the two different Pic treatments provided results similar to MB 40 PE in terms of *R. solanacarum* control after fumigation, plant growth at 15 WAT, plant height, plant mortality, root disease index and ginger yield at the end of the trials. The results confirmed that the two tested Pic treatments both provide an effective alternative to MB. However, based on our present field results, it is too soon to say which one of the Pic treatments is better, and more studies are needed in order to draw a definitive conclusion on this point. Generally speaking, the temperature must be between 4.4 to 26.7°C (40 to 80°F) at the depth of injection when Pic is applied [25]. In our filed trials, when Pic was applied, the soil temperatures at 5 cm depth during the day of two different Pic treatments (in November 2012 and in March 2013) was about 15 to 21°C and 13 to 19°C, respectively (**Figure 1**). In November 2012 treatments, with the winter coming, the soil temperature will become lower and lower, and the lowest soil temperature will be about 0°C. Although from previous laboratory results, it would appear that chloropicrin is effective against *Verticillium* at low temperatures around 50°F±2°F (8.9 to 11.1°C) [26]. In the field application, whether and how low temperatures affect the effect of Pic on *R. solanacarum* is still unclear to us.

The soils in our three field trials were heavily infested by *R. solanacarum*, but at 9 WAT the fumigation treatments did not significantly affect ginger germination levels except for MB 40 PE treatments in trial III. This indicates that ginger bacterial blight did not generally appear in the initial growth stage of ginger, and this could be attributed to the relatively low temperature, low rainfall patterns and low soil moisture during the initial growth stage.

Pic is a strong eye irritant and has a pungent unpleasant smell, which can pose barriers to its adoption. Well-equipped commercial companies generally provide safe soil fumigation services in the United States, Europe, or Japan. However, soil fumigation in China is mostly conducted by individual farmers who generally lack essential application tools and personal protection equipment. Considering the environment emissions and potential human exposure, the gelatin capsule (gel cap) formulation of Pic will offer a promising solution [10], [27]. The use of TIF would allow greater retention of Pic than the conventional PE film, trapping the fumigant for a longer period near the soil surface and thereby increasing the dose and prolonging the exposure of soilborne pathogens to the fumigant [28], [29]. In the present field trials, Pic 50 TIF treatments applied in November were generally similar or even superior to Pic 50 PE treatments applied in March for controlling *R. solanacarum* and improving plant growth and ginger yield. However, we are not able to attribute this to the use of TIF before removing the effect of the different application seasons. Pic is normally applied in the spring (during March and April) about one month before ginger is transplanted in the field. The soil temperature is low (about 8 to 18°C during the day) during this fumigation period, so it would be desirable to examine in more detail the feasibity of applying Pic in the autumn (during October and November, just after the ginger harvest) when soil temperature is relatively high (about 15 to 25°C during the day).

In summary, our studies determined that the fumigant Pic had similar efficacy to MB in terms of controlling ginger bacterial blight caused by *R. solanacarum*, ginger germination, plant height, plant mortality, number of leaves per tiller and ginger yield. However, more detailed work to identify the most suitable application methods (formulation, rates, and application seasons) and appropriate combinations [30] with other fumigants or biological agents (for example *Trichoderma asperellum*, *Bacillus subtilis* or others) is necessary before Pic treatments can be recommended as an efficient alternative to methyl bromide for ginger production in China.
